# Nitrogen fixation and other biogeochemically important features of Atacama Desert giant horsetail plant microbiomes inferred from metagenomic contig analysis

**DOI:** 10.1093/aob/mcac060

**Published:** 2022-05-09

**Authors:** Anchittha Satjarak, Linda E Graham, Marie T Trest, Joy Zedler, Jennifer J Knack, Patricia Arancibia-Avila

**Affiliations:** Plants of Thailand Research Unit, Department of Botany, Faculty of Science, Chulalongkorn University, Bangkok, Thailand; Department of Botany, University of Wisconsin-Madison, Madison, WI, USA; Department of Botany, University of Wisconsin-Madison, Madison, WI, USA; Department of Botany, University of Wisconsin-Madison, Madison, WI, USA; Department of Botany, University of Wisconsin-Madison, Madison, WI, USA; Department of Basic Sciences, University of Bío-Bío, Chillan, Chile

**Keywords:** Biogeochemistry, *Equisetum xylochaetum*, metagenome, plant microbiome, vanadium nitrogenase, wetlands

## Abstract

**Background and Aims:**

Canyon stream beds in the hyperarid Atacama Desert surprisingly harbour magnificent groves of endemic giant horsetail wetland plants, *Equisetum xylochaetum*. Our previous metagenomic study of eukaryotes closely associated with this plant indicated that the microbiome included prokaryotes that might likewise influence host success and environment. We explored this possibility by using the metagenomic sequence to characterize prokaryote taxa and functional genes present in the microbiome of *E. xylochaetum* sampled from remote sites differing in the degree of anthropogenic disturbance. We focused on biogeochemical functions known to be important in wetland ecosystems.

**Methods:**

To ensure that analyses were conducted on microbes most closely associated with plants, we extracted DNA from well-washed plant organs whose microbial biofilms were revealed with scanning electron microscopy. To assess the benefits of longer sequences for taxonomic and gene classifications, results of analyses performed using contigs were compared with those obtained with unassembled reads. We employed methods widely used to estimate genomic coverage of single taxa for genomic analysis to infer relative abundances of taxa and functional genes.

**Key Results:**

Key functional bacterial genera (e.g. *Hydrogenophaga*, *Sulfuritalea* and *Rhodoferax*) inferred from taxonomic and functional gene analysis of contigs – but not unassembled reads – to occur on surfaces of (or within) plants at relatively high abundance (>50× genomic coverage) indicated roles in nitrogen, sulfur and other mineral cycling processes. Comparison between sites revealed impacts on biogeochemical functions, e.g. reduced levels of the *nifH* gene marker under disturbance. Vanadium nitrogenases were more important than molybdenum nitrogenases, indicated by both functional genes and taxa such as *Rhodomicrobium* and *Phaeospirillum* inferred from contigs but not unassembled reads.

**Conclusions:**

Our contig-based metagenomic analyses revealed that microbes performing key wetland biogeochemical functions occur as tightly adherent biofilms on the plant body, not just in water or sediments, and that disturbance reduces such functions, providing arguments for conservation efforts.

## INTRODUCTION

Global wetlands currently occupy an estimated area of at least 12.1 × 10^6^ km^2^, of which about half is continuously wet and the rest intermittently so, most being inland freshwaters vulnerable to climate change impacts (Davidson *et al.*, 2018). Wetland bacteria are known to play important roles in modern global nitrogen (N), sulfur (S) and carbon (C) cycling and metal transformations, and wetland plants produce organic exudates and oxygen that influence these processes (Lamers *et al.*, 2012). As particularly close plant–microbe associations, plant microbiomes are widely understood to have ecosystem-level impacts on mineral cycling. For example, N-fixing bacteria (rhizobia) aid plant nutrition (Stanton *et al.*, 2019), thereby influencing both N cycling and C cycling, by increasing primary production and sequestration of plant-generated organic C.

Plant microbiomes – defined as host-associated communities of prokaryotic species, eukaryotic microbiota and their genes – improve mineral nutrient availability or otherwise benefit plant health (Rodriguez *et al.*, 2019; Wozniak *et al.*, 2019). Plant microbiota are known to include heritable components (e.g. Walters *et al.*, 2018). Mechanisms of vertical inheritance include passage from one plant generation to the next via reproductive structures (Nelson, 2018) and genetically controlled production of exudates that influence microbiome assembly (Stringlis *et al.*, 2018; Huang *et al.*, 2019; Pascale *et al.*, 2020). Metagenomic sequence data have been employed to assess the taxonomic composition and potentially beneficial functions (e.g. nitrogen fixation) of microbiomes of diverse modern representatives of long-enduring algal or non-vascular plant clades (Graham *et al.*, 2018). Here, the focus is upon a species of the globally widespread plant *Equisetum*, commonly known as horsetail or scouring rush, considered the world’s oldest vascular plant genus. Molecular diversification (timetree or molecular clock) analyses indicate an Early Carboniferous [343 million years ago (mya)] stem age and a crown age of Middle Jurassic (170 mya) for the genus *Equisetum* (Christenhusz *et al.*, 2021). Later Carboniferous (304.2 mya) stem age and mid-Mesozoic crown age were indicated by molecular data combined with fossil information (Elgorriaga *et al.*, 2018).

*Equisetum xylochaetum*, recently segregated from *E. giganteum* (Christenhusz *et al.*, 2019), is endemic to Atacama Desert valley streambeds of southern Peru and northern Chile, where the plant forms magnificent groves. The extensively branched stems of *E. xylochaetum* are known to reach 4.0 cm in diameter and more than 4 m in height, and produce extensive and deep below-surface rhizome/root systems (Husby *et al.*, 2011; Husby, 2013), regarded as key to plant survival in stressful environments and long-term clade endurance. A previous metagenomic study with a focus on eukaryotic components of the *E. xylochaetum* microbiome, which reported the occurrence of associated fungi though not known mycorrhizae, indicated that bacteria were also closely associated with the plant host (Satjarak *et al.*, 2021), but did not characterize the bacteria or their genes in terms of taxonomy or biogeochemical function. Other *Equisetum* species have been linked to N fixation or other biogeochemical functions considered key to other wetland systems (Uchino *et al.*, 1984; Marsh *et al.* 2000). Here, we report the results of shotgun metagenomic sequencing approaches to identifying and estimating abundances of bacterial taxa and genes associated with *E. xylochaetum*, with a focus on biogeochemical functions.

One goal of this study was to consider if the *E. xylochaetum* microbiome might include bacteria that help plants, by fixing N or in other beneficial ways, to cope with its stressful hyperarid environment (Arroyo *et al.* 1988). With the exception of El Niño-associated rain events (Chávez *et al.*, 2019; Ortega *et al.*, 2019), annual rainfall along the northern Chilean coast is typically 0.5–0.6 mm (Arica and Iquique weather stations), associated with sub-tropical high pressure, a cold offshore current and wind. Evaporation and atmospheric deposition, coupled with little erosion over millions of years, has led to surface accumulations of gypsum, anhydrites, halite, perchlorates, chlorides, iodates and nitrates (Michalski *et al.*, 2004; Voight *et al.*, 2020). The Atacama Desert is notable for the world’s only natural nitrate deposits (saltpeter and caliche) that formed over 10 million years (Reich and Bao, 2018), and were intensively mined until the industry collapsed with the rise of industrial N fixation. During the mining period >100 000 acres (approx. 40 470 ha) of desert were impacted by the activities of >100 processing facilities, some of which might have impacted plant populations or their microbiomes. To gain insight into possible anthropogenic impacts (such as mining) on *E. xylochaetum* microbiomes, we compared the microbiomes of plants growing in sites differing in their degree of disturbance.

## MATERIALS AND METHODS

### Plant sampling

Plant tissue samples were collected from both remote and more accessible Atacama valley stream bed sites. Sampled sites were among several mapped localities that had previously been sampled for physiological studies focused on giant *Equisetum* adaptations to elevated salinity conditions arising from high levels of evaporation (Husby *et al.*, 2011, 2014; Husby, 2013). Sites were located in the Tarapacá region, with similar mid-day temperatures during mid-January sampling (30–31 °C) and sandy soils, but differed in altitude, in remoteness and in present and past levels of human disturbance.

The remote Huasquiña (also spelled Guasquiña) sampling site, here abbreviated HUA, was located at an altitude of 1983 m, at GPS co-ordinates: –19.752535, –69.404068. This site was located 18.3 km from Chile Highway 15, along the A-495 road, which included a steep single-lane switchback ending at the small village of HUA. The sampling site was about 1 km distant from the village, occupied by a small population of indigenous Aymara descendants. Remains of ancient agricultural terraces were observed at the sampling site, but not modern crops.

To gain insight into possible effects of human disturbance on our samples, we also sampled a more accessible Chiza Valley (CHI) site located 166 km away from HUA and approx. 100 m from Highway 5, Chile’s main north–south highway. The accessible site, altitude 350 m, was located at GPS co-ordinates: –19.198696, –70.043582. This area has been disturbed not only by modern vehicular traffic, but also by past nitrate mining (Marr, 2007).

### Sample collection and processing

Samples were taken from both above-ground shoots and below-ground plant parts from moist soil at a depth of about 0.3 m at both sites. Collectors used nitex gloves and new implements to reduce the potential for sample contamination. At HUA, three replicate pieces a few centimetres in length were taken aseptically from *E. xylochaetum* upper and lower above-ground stems, rhizomes and roots, plus one sample of internal tissue at a stem joint, for storage in 90 % ethanol, for later DNA extraction. Ethanol is known to preserve DNA quality when used during plant collections at remote sites (Bressan *et al.*, 2014). At CHI, three replicates of upper and lower stems and roots were likewise collected for DNA extraction (at this site rhizomes were not encountered during excavation to a 1 m depth). Samples collected for scanning electron microscopy (SEM) imaging were preserved in freshly prepared 2 % glutaraldehyde in 0.1 m pH 7 phosphate buffer, then washed three times in buffer and dehydrated in an ethanol series. Materials were then critical point-dried and coated with iridium prior to study with a Hitachi S-4800 ultra-high-resolution cold cathode field emission scanning electron microscope operated at 5 kV (Satjarak *et al.*, 2021). Herbarium specimens were archived as CONC-CH 6005 at the University of Concepción herbarium (Conc.), Concepción, Chile.

### DNA extraction, metagenomic sequencing and annotation

Plant samples were washed in three changes of sterile deionized water prior to DNA extraction to remove ethanol, maximize the sampling of closely adherent microbial biofilms and minimize the inclusion of loosely attached soil microbes. Detailed methods employed for washing, low-shear DNA extraction, DNA transport, sample pooling, Illumina HiSeq 2500 metagenomic sequencing, sequence quality checking, contig assembly and checking, SILVAngs 1.3 (Quast *et al.*, 2012) classification of contigs and estimating abundances of rRNA genes and functional genes in contigs were the same as described in our previous publication on eukaryotic members of the *E. xylochaetum* microbiome, which employed the same metagenomic dataset (Satjarak *et al.*, 2021). Technical replicates (including above- and below-ground tissues) for each of the two sites were pooled in order to achieve DNA quantities needed for metagenomic sequencing.

Innovative technical features included the use of DNA extraction procedures designed to yield larger shotgun sequencing fragments to reduce the likelihood for artefact sequence formation during downstream contig assembly. Specifically, DNA extraction done using the MoBio PowerSoil kit (Qiagen, Hilden, Germany) employed a modified lysis procedure designed to reduce DNA shearing, as suggested by Qiagen technical personnel (pers. comm.). To reduce DNA shearing, samples were vortexed for only a few seconds prior to heating at 70 °C for 5 min, a process that was then repeated. We assembled reads into contigs to acquire longer sequences before submitting the sequence to taxonomic or gene classification pipelines, with the goal of increasing the accuracy of bacterial and gene classifications (Satjarak *et al.*, 2021). The assembler used paired reads to detect and resolve chimeric contigs produced from misassembly; low numbers of chimeric sequences were detected and removed from the *E. xylochaetum* metagenomic dataset (Satjarak *et al.*, 2021). To shed light on the effects of using contigs as input to platforms for identification of bacterial taxa, in the current study we compared results obtained when contigs were employed vs. results from the use of unassembled reads, as is more commonly done.

The number of raw shotgun reads obtained for the remote HUA site was 96 426 246, and a similar number of 101 656 742 reads was obtained for the accessible CHI site, justifying comparisons of results obtained for the two sites. Raw sequences were transferred to the MG-RAST online server (Meyer *et al.*, 2008) for removal of sequencing adapters using Trimmomatic v 0.33 (Bolger *et al.*, 2014), removal of artefactual sequences and filtering of ambiguous bases. Sequences passing these quality control steps were annotated using BLAT (Kent, 2002) against M5NR (Wilke *et al.*, 2012), which contained non-redundant sequences of protein and ribosomal databases.

### Taxonomic annotations

Taxonomic annotations using rRNA-encoding genes within contigs as input to the SILVAngs taxonomic platform were accomplished as described in a previous publication describing eukaryotic members of the *E. xylochaetum* microbiome (Satjarak *et al.*, 2021). Taxonomic annotations performed from unassembled reads were done by comparing sequences with the SILVA SSU and SILVA LSU databases using the parameters ‘percent identity’ = 97, ‘evalue’ = 1-e-5, ‘alignment length = 15 and ‘abundance’ = 10. To compare results obtained by taxonomic analysis of contigs with those based on unassembled reads, we assessed the degree to which the analyses detected bacterial genera for which functional gene annotations indicated significant roles in N or S cycling. Raw sequence data were deposited in Bioproject PRJNA555713. Sequence data for the remote HUA site are in accession SRX6486517, and sequence data for the more accessible CHI site are in accession SRX6486516.

### Functional gene annotations

Functional gene annotations were performed for genes known to be associated with key wetland biogeochemical functions, particularly nitrogen fixation, denitrification, and S reduction and oxidation. To investigate the presence of relevant protein-coding sequences, an annotation step was carried out using the SEED Subsystem and KEGG (Kyoto Encyclopedia of Genes and Genomes) Orthology databases (Kanehisa *et al.*, 2017). To filter and visualize the functional annotations, we used the analysis function implemented in the MG-RAST server using KEGG. To illuminate the taxonomic relationships (microbial sources) of the protein-coding sequences of genes involved in these metabolisc pathways, we searched for protein-coding genes of interest in the assembled contigs having length longer than 10 000 bp. We annotated the contigs using the RAST server https://rast.nmpdr.org (Aziz *et al.*, 2008), using the following parameters: Annotation scheme ClassicRAST; Preserve gene calls, no; Automatically fix errors, yes; Fix frameshifts, yes; Backfill gaps, yes; and Domain, Bacteria (so no eukaryotic genes were annotated). Pathway mapping was done using the KAAS (KEGG Automatic Annotation Server) available at https://www.genome.jp/kegg/kaas/, using the following parameters: Metagenomes; KAAS job request (SBH method for amino acid sequence query); Search program: GHOSTX (amino acid query only); GENES dataset, for Genes; Assignment method: SBH (single-directional best hit) (Moriya *et al.*, 2007).

### *Comparison of microbiota associated with Chilean* E. xylochaetum *and Chilean peat moss*

We had previously reported the results of a similar metagenomic study of a Chilean peat moss (Graham *et al.*, 2017), for which raw sequence data are archived in the NCBI Short Read Archive (http://www.ncbi.nlm.nih.gov/sra) as BioProject PRJNA 384422. Similarities in washing and other methods facilitated a comparison of bacterial species closely associated with the peat moss and *E. xylochaetum*, because results might contribute to future efforts to determine if there exists a core assemblage of bacteria associated with wetland plants.

## RESULTS

### Bacterial diversity and estimated abundances

**Fig. 1.**
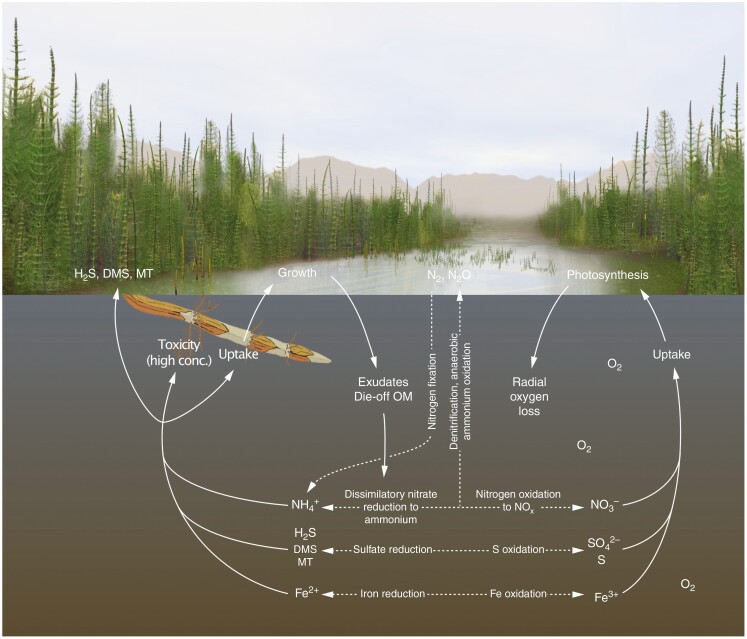
Present and potential deep time wetland sphenophyte–microbe interactions related to N, S and Fe biogeochemistry. Digital painting by L. Wilcox, incorporating biogeochemical information from Lamers *et al.* (2012).

Our metagenomic study revealed that bacterial taxa and genes related to biogeochemical processes known to play important roles in modern wetlands (Fig. 1) occur in the microbiomes of Atacama Desert plants having structural features consistent with *Equisetum xylochaetum* (Supplementary data Fig. S1). Differences were observed when input to the bacterial taxonomic platform (Supplementary data Fig. S2) was in the form of contigs (Supplementary data Fig. S3) vs. the use of unassembled read data (Supplementary data Fig. S4). When contigs were used as taxonomic platform input, total bacterial diversity in the *E. xylochaetum* microbiome, inferred using a detection criterion of 16S and/or 23S rRNA gene sequences present with at least 10× mean sequence coverage, included 90 bacterial genera. In contrast, when unassembled reads were used as input to the same taxonomic platform, using a detection criterion of at least ten reads, 312 bacterial genera were detected (Supplementary data Fig. S5).

**Fig. 2.**
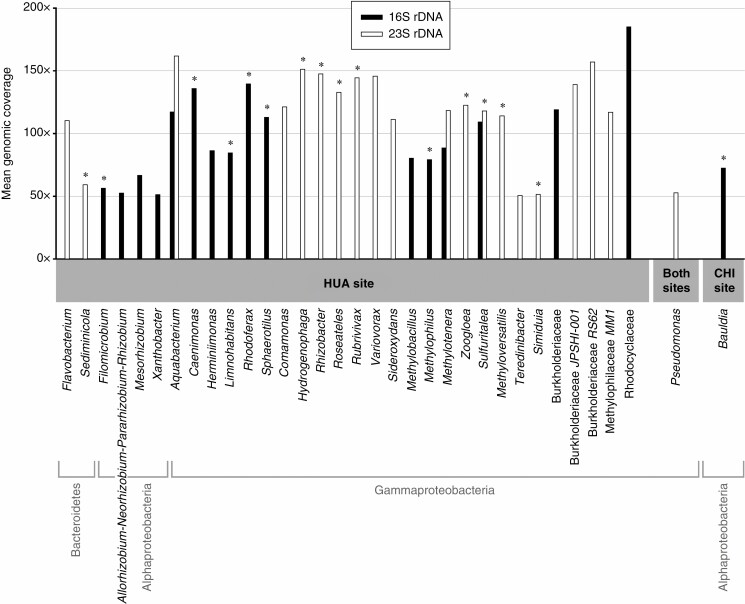
Bacterial taxa inferred by taxonomic analysis of contigs to occur in relative abundance (>50× genomic coverage) at a more remote/less disturbed site (HUA) vs. a more disturbed site (CHI) by 16S and/or 23S rRNA gene sequences. Asterisks indicate taxa not detected in an analysis of unassembled short reads, emphasizing the value of employing longer contigs for taxonomic analyses.

Relatively abundant bacterial genera and higher taxa that were present in the *E. xylochaetum* microbiome were identified by employing a criterion of at least 50× mean coverage of 16S and/or 23S rRNA gene sequences in contigs (Fig. 2) or a criterion of at least 50 reads for unassembled read data (Supplementary data Fig. S6). When the results of the two taxonomic processes (contigs vs. unassembled reads as input) for these relatively abundant genera were compared, some generic overlap was observed, *Rhizobium* being one example. However, 16 of the relatively abundant genera identified by contigs (indicated by asterisks in Fig. 2) were not detected in the analysis of unassembled reads, even those present in low abundance. This difference is important because the 16 relatively abundant taxa observed only by contig taxonomic analysis (see Fig. 2) included genera commonly associated with wetland transformations of N, S and iron (Fe) for which we identified contigs containing homologous functional genes (Supplementary data Fig. S7). Examples of key functional, relatively abundant genera not indicated by any unassembled reads include *Rhodoferax*, *Hydrogenophaga* and *Sulfuritalea*, yet we found homologous N, S or Fe metabolism genes for each of these genera. In contrast, functional gene analysis did not reveal key functional features uniquely associated with any bacterial genera found only by analysis of unassembled short reads.

Several bacterial genera (*Aquabacterium*, the denitrifier *Methylotenera* and the S-oxidizing chemolithotroph *Sulfuritalea*) were inferred to be relatively abundant based on contig analysis of both 16S and 23S rRNA gene mean sequence coverage at the >50× level (see Fig. 2). Other relatively abundant bacterial genera were indicated by either 16S or 23S rRNA gene-containing contigs, but not both (see Fig. 2). For example, in the contig analysis, the Fe(III) reducer *Rhodoferax* was detected only by the 16S rRNA gene marker. The Fe(II) oxidizer *Sideroxydans* was detected only by the 23S rRNA gene marker (see Fig. 2), a result consistent with the use of unassembled short reads.

Bacterial genera that were inferred to be relatively abundant by contig-based taxonomic analysis, but that were not detected by any reads when unassembled reads were used as taxonomic platform input, are indicated by an asterisk in Fig. 2. This data display also indicates that particular bacterial genera known for involvement in N, S and Fe transformations (e.g. *Rhodoferax*, *Hydrogenophaga* and *Sulfuritalea*) are relatively abundant at the less disturbed HUA site, but not at the CHI site impacted by mining and other anthropogenic effects.

At least 37 bacterial taxa detected at the two sites by 16S and/or 23S rRNA contigs having mean coverage levels of at least 10× could not be identified to genus with the use of current databases that are primarily based on cultured microbes (Supplementary data Figs S8 and S9), suggesting the occurrence of new, as yet uncultured biodiversity. Among these new taxa, five that were classifiable to the family level (such as Burkholderiaceae) were relatively abundant, indicated by at least 50× mean genomic coverage (see Fig. 2).

**Fig. 3.**
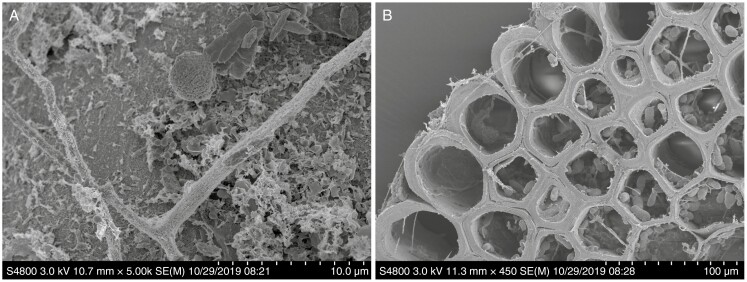
Scanning electron microscopy imaging of *Equisetum xylochaetum* and associated microorganisms. (A) Root surface showing the microbial biofilm. (B) Root cross-section showing the surface microbial biofilm.

The SEM of well-washed underground plant tissues revealed extensive coverage by microbial biofilms (Fig. 3). These images document the close attachment to plant surfaces of microbes having diverse morphotypes.

### *Differences in* E. xylochaetum *microbiota at sites contrasting in disturbance*

Both taxonomic analysis procedures (use of contigs and unassembled short reads) indicated that the microbiota of *E. xylochaetum* sampled from the two study sites contained many common genera. However, both types of analysis indicated that the less disturbed, more remote HUA site featured more plant microbiome bacterial diversity than did the more disturbed, less remote site CHI. This diversity difference between the two sites is not due to a difference in metagenomic sequence amounts, because the CHI site plants yielded >5 million more sequence reads than did plants at HUA.

### Equisetum xylochaetum *microbiome genes linked to N cycling*

**Fig. 4.**
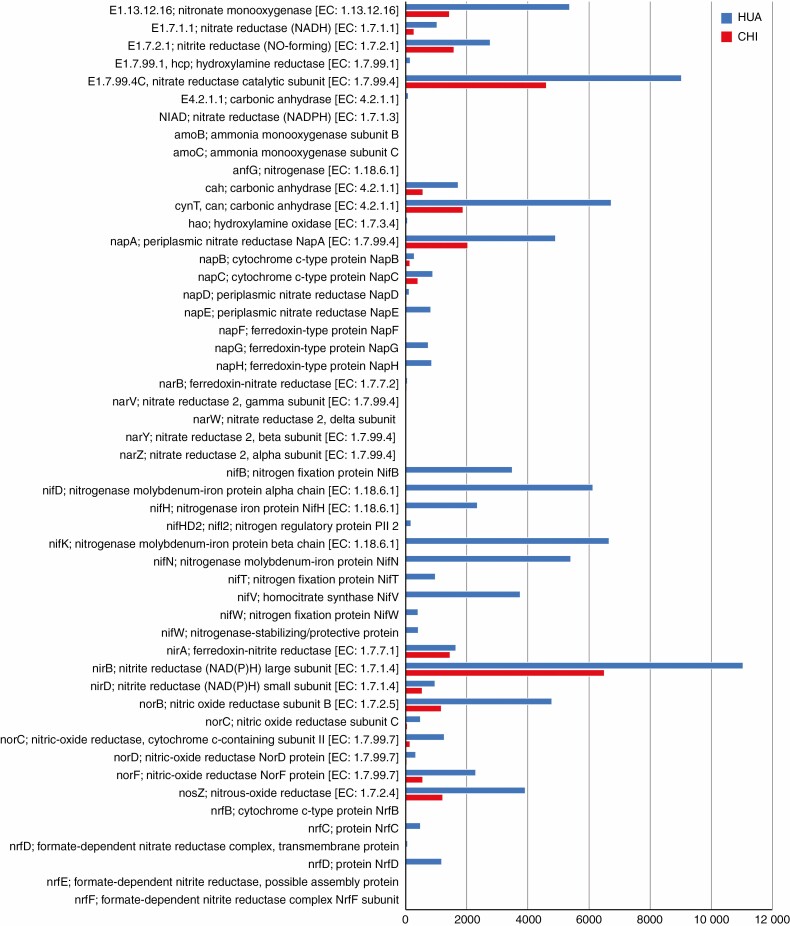
Relative abundances of protein-coding genes involved in N metabolism in plant microbiomes at two sites contrasting in disturbance level. Numbers are unassembled reads mapping to each gene type. In all cases, N metabolism genes were more abundant at the more remote, less disturbed site (HUA) (blue bars at tops of paired bars, or single bars) than at the more accessible, more disturbed site (CHI) (red bars at bottoms of paired bars). None of the N metabolism genes listed was only present at the more disturbed CHI site.

The KEGG pathway analysis indicated that both study sites possessed *E. xylochaetum* microbiome genes involved in N and S metabolism (Supplementary data Figs S10 and S11). Comparisons of N metabolism-related gene abundances (Fig. 4) indicated more evidence for N fixation at the remote, less disturbed HUA site. For example, *nifH*, commonly used as a marker for N fixation, was distinctly more abundant in the microbiome of plants at HUA. The HUA site was also notable for relatively abundant N-fixing genera such as *Rhizobium*, *Mesorhizobium* and *Xanthobacter*, detected by both contig and unassembled read analyses. Nitrogen fixation gene annotation revealed that one long contig from a gammaproteobacterial taxon present at HUA contained most genes known to be associated with N fixation (Supplementary data Fig. S12). The more accessible, more disturbed CHI site may have been less N limited, possibly as the result of past nitrate mining activities that might have increased nitrate availability to plants. Nitrate reductases were indicated for *E. xylochaetum* microbiomes at both sites (see Fig. 4).

The remote, less disturbed HUA site was the source of sequences encoding both molybdenum (Mo) and vanadium (V) nitrogenase subunits, with contigs encoding V nitrogenase subunits inferred to be present in higher estimated genomic coverage levels than those encoding Mo subunits. Two types of V nitrogenase sequence were inferred for the remote HUA site. The contig k119_56099 (length = 41 985 bp) contained four full-length protein-coding sequences: nitrogenase (vanadium–iron) alpha chain: vnfD (EC 1.18.6.1); nitrogenase (vanadium–iron) beta chain: vnfK (EC 1.18.6.1); nitrogenase (vanadium–iron) delta chain: vnfG (EC 1.18.6.1); and nitrogenase (vanadium–iron) cluster gene Avin0834, and nitrogenase vanadium co-factor synthesis protein VnfY. A second contig k119_208769 (length = 80 315 bp) included one full-length gene annotated as nitrogenase vanadium co-factor synthesis protein VnfX, of average genome coverage 24.23× and maximum coverage 48×.

The purple non-sulfur bacterial genera *Rhodomicrobium* and *Phaeospirillum* inferred by contig analysis (but not from unassembled reads) to occur in the *E. xylochaetum* microbiome are among bacterial types reported to produce V nitrogenases as well as Mo nitrogenases. *Rhodomicrobium* was detected by both 16S and 23S rRNA gene markers and only at the remote HUA site, whereas *Phaeospirillum* was detected only by 23S rRNA genes and only at the accessible CHI site.

The KEGG analysis also indicated the presence of *Equisetum* microbiome genes associated with nitrification, nitrate reduction and denitrification pathways at both sample sites. For all N cycle genes detected, abundance was higher at the remote, less disturbed HUA site than the more disturbed CHI site (see Fig. 4). This result did not derive from a difference in total amount of metagenomic sequence, because more sequence was acquired for the CHI site.

### Equisetum xylochaetum *microbiome genes linked to S cycling*

**Fig. 5.**
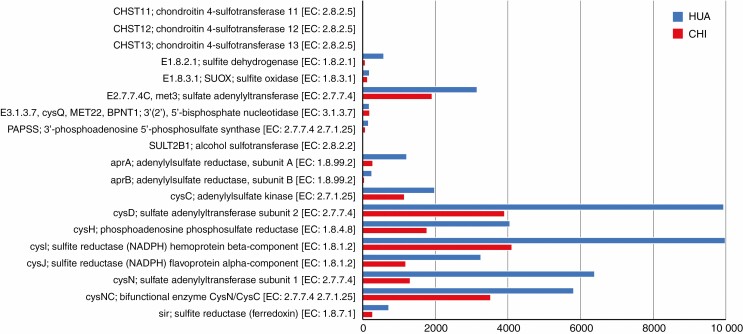
Relative abundances of protein-coding genes involved in S metabolism in plant microbiomes at two sites contrasting in disturbance level. Numbers are unassembled reads mapping to each gene type. In all cases, S metabolism genes were equal to or more abundant at the more remote, less disturbed site (HUA) (blue bars at tops of pairs of bars). None of the S metabolism genes listed was only present at the more disturbed CHI site.

The KEGG analysis indicated the presence of similar S reduction and oxidation pathway genes in the *Equisetum* microbiome at the two sample sites. For all S cycle genes detected, abundances were equal to or higher at the lower disturbance site HUA in comparison with the higher disturbance site CHI (Fig. 5). This result did not derive from a difference in total metagenomic sequence, which was higher at the CHI site.

**Fig. 6.**
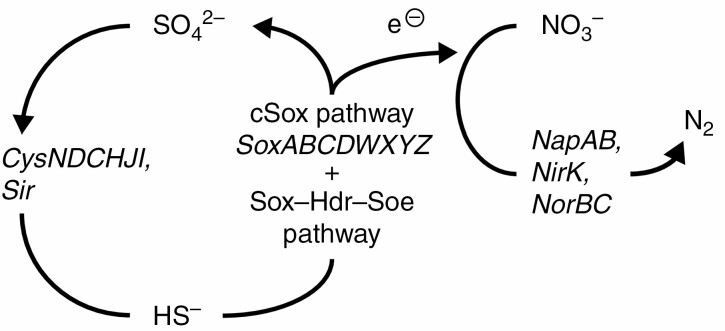
Metabolic linkage of N and S metabolism. Bacterial genes involved in S and N metabolism processes that were detected by contig analysis in the microbiomes of Atacama Desert *Equisetum xylochaetum*.

Because S oxidation closes the S cycle, in the process generating electrons used in denitrification (Fig. 6), S oxidation is considered a particularly important biogeochemical process. Consequently, we annotated contigs containing *hdrA*, *sox*, *dsr* and *soe* genes associated with S oxidation or other S metabolism pathways that were present in the *E. xylochaetum* microbiome. *SoxABCDXY* genes with full protein-coding regions were present on a single contig (classifying as HGW-Betaproteobacteria-4) at remote HUA, and a contig containing full-length sequences of *SoxABCDXYZW* (classifying as Kiloniellaceae) was present at the accessible CHI site. SoxXYZAB proteins are characteristic of all three known S oxidation pathways. Gene annotations also suggested the occurrence at both sites of the Sox–Hdr–Soe pathway, in which Dsr proteins are replaced by Hdr proteins. Even so, full-length SoeABC genes, classifying as Kiloniellaceae, were detected only at the accessible CHI site.

Annotation of genes associated with S metabolism, namely *Cys* and *Sir*, yielded information consistent with ribosomal marker evidence for particular, abundant bacterial genera at remote HUA (see Fig. 2). These included *Aquabacterium*, *Rhodoferax*, *Hydrogenophaga*, *Methylotenera* and *Flavobacterium*, the latter also associated with the denitrification gene *NorBC.* At the accessible CHI site, annotation of genes associated with S reduction and denitrification indicated that these functions are performed by uncultured taxa.

### *Comparison of microbiome taxa and functions inferred for* E. xylochaetum *with those inferred for non-vascular wetland plants*

Bacterial taxa classified by using contigs and functions associated with *E. xylochaetum* plants growing at the remote HUA site were compared with those previously reported for Chilean *Sphagnum fimbriatum*, a seedless plant whose microbiota we had previously assessed using similar washing and DNA extraction techniques, the same classifier and the same detection criteria. The comparison revealed evidence for several common microbial taxa and functions related to wetland biogeochemistry or plant health: *Magnetospirillum* associated with Fe(III) reduction, *Sulfurospirillum* (S reduction, denitrification), *Sulfuritalea* and *Thioalkalivibrio* (chemolithothrophy, S oxidation), *Hydrogenophaga* (H oxidation), *Methylotenera* (denitrification), *Rubrivivax* (anoxygenic photosynthesis), *Mesorhizobium* and *Rhizobium* (N fixation, and plant growth promotion (*Variovorax* and *Rhizobacter*).

## DISCUSSION

### The value of analysing microbiomes tightly associated with plant bodies

Together, our microscopy and molecular results, based on analysis of DNA extracted from well-washed plant samples, indicate that microbial taxa associated with key wetland biogeochemical processes are closely associated with wetland plant bodies, not just present in water or sediments. In contrast to our use of shotgun metagenomic sequence data to focus on microbiomes tightly adherent to plant bodies, other studies often report the use of amplicons to characterize the microbiota of soils in contact with plant roots, i.e. the rhizosphere. For example, Astorga-Eló *et al.*, (2020) used 16S rRNA gene amplicons to study soil microbiota associated with roots of the Atacama Desert flowering plant *Cistanthe* sp. Differences in research design and methodology make it difficult to compare the results of such studies with our metagenomic analysis of well-washed plant materials. In view of evidence that plant organic secretions and oxygen affect wetland biogeochemical processes (e.g. Lamers *et al.*, 2012) and that the *Equisetum* rhizome and roots provide extensive below-ground surface area for microbial attachment, our observations indicate the value of studying microbiota closely associated with horsetails and other wetland plants.

### The value of using multiple taxonomic markers and contigs for inferring microbial diversity

Although plant microbiome studies often employ 16S rRNA genes as taxonomic markers, the differences we found in results obtained with 16S rRNA vs. 23S rRNA gene markers indicate the value of employing both markers to maximize detection of bacterial diversity in plant microbiomes. We also observed different results when we employed contigs as input to the taxonomic platform vs. the use of unassembled read data, as is more common. Some of this difference may result from a year time gap between the analyses, because some new taxa were probably added to the taxonomic database during that period. Another explanation for the difference may have resulted from the higher stringency of the process by which longer contigs had to match database sequences at the 97 % level. Some of the genera inferred only from short unassembled reads may have possessed rRNA genes that would have lacked homology had they been compared with database reference sequences along a greater sequence length.

Inspection of our results, including considerations of relative abundance, provides evidence that the use of contigs (rather than short unassembled reads) may be particularly important when considering functional roles of the microbiome. For example, bacterial genera such as *Hydrogenophaga*, *Sulfuritalea* and *Rhodoferax* inferred from ribosomal and functional gene analysis of contigs (but not unassembled reads) were associated with plants at >50× genomic coverage, indicating key roles in N, S and other cycling processes. Also, the purple non-sulfur bacterial genera *Rhodomicrobium* and *Phaeospirillum* that we inferred by contig analysis (but not from unassembled reads) to occur in the *E. xylochaetum* microbiome are among bacterial types reported to produce V nitrogenases as well as Mo nitrogenases (Harwood, 2020).

### *Roles of* E. xylochaetum*-associated bacteria in key wetland biogeochemical cycling processes*

Although we found taxonomic and functional gene evidence for the presence of Mo nitrogenases, V nitrogenases were more abundant in the microbiomes of *E. xylochaetum* that we sampled. Because Mo is the scarcest micronutrient in Earth’s crust, its availability can limit biological N fixation, indicating one advantage of V nitrogenases, as V concentrations are up to 200× higher in soils than those of Mo (Bellenger *et al.*, 2020). Because V nitrogenases are known to generate more H_2_ per N_2_ than do Mo nitrogenases, the former may provide redox relief by decreasing excess reducing power.

Our finding of plant microbiome genes associated with nitrification, nitrate reduction and denitrification was consistent with evidence for extensive colonization of the sandy soils by aerenchymatous *Equisetum* rhizomes, as such sites are unlikely to experience extended periods of hypoxia known to inhibit nitrification and reduce denitrification (Jäntti *et al.*, 2021). Our study also detected genes such as *hdrA*, *sox*, *dsr* and *soe* associated with S oxidation or other S metabolism pathways (Watanabe *et al.*, 2014, 2017, 2019), though our observations suggested that S oxidation and S reduction may be performed by different bacteria at the two sites. Future work might determine if this difference is a possible effect of anthropogenic disturbance at CHI, such as past mining activities.

### *Comparison of Chilean peat moss microbiomes with those of* E. xylochaetum

Our comparison of microbiomes of giant Atacama Desert horsetails with those of a Chilean peat moss that had been washed and otherwise processed using similar methods (Graham *et al.*, 2017) revealed several common features, including bacteria known to foster plant health. These included nitrogen fixers and plant growth promoters. However, unlike peat mosses, which are commonly associated with methane-oxidizing bacteria that contribute importantly to global carbon cycling (Alexandrov *et al.* 2020), methanotrophic bacteria were not detected in the microbiomes of Atacama Desert *E. xylochaetum*. We note that *E. fluviatile* in northern hemisphere lakes is reportedly associated with methane oxidation (Kankaala and Bergström, 2004), indicating possible variability in this biogeochemical function. Because modern *Equisetum* is globally widespread, and encompasses at least 18 species (Christenhusz *et al.*, 2019), more effort is needed to understand how wetland *Equisetum* may be involved in wetland methane dynamics. Additional metagenomic analysis might reveal evidence for association of methanotrophs with *Equisetum*. Future comparative microbiome studies might indicate if a core *Equisetum* microbiota or core wetland plant microbiota exist.

In summary, our metagenomic sequencing data from the remote Atacama Desert reveal that microbiomes of giant horsetail plants include bacterial taxa and genes that indicate globally significant effects on wetland N and S cycling and transformations of other materials, and may also foster plant survival in stressful environments. Our evidence for differences in plant microbiomes sampled from remote, less disturbed vs. more accessible, more disturbed sites indicates anthropomorphic effects on microbiome functions and emphasizes the need to protect biotic communities at remote locations. Future disturbances of *E. xylochaetum* and its microbiomes could arise from renewed mining of Atacama Desert nitrate deposits for extraction of valuable iodine (Reich and Bao, 2018) or climate changes that affect the amount of Andean snowmelt that feeds Atacama Desert rivers and streams. Considering these threats, a programme of microbiome surveillance might be useful for monitoring the sustainability of ecological services provided by populations of *E. xylochaetum*. Future studies could possibly explore microbiome variation that might occur in association with underground parts sampled at differing depths. Additional studies might also be conducted of the microbiomes of other *Equisetum* species in other habitats.

## SUPPLEMENTARY DATA

Supplementary data are available online at https://academic.oup.com/aob and consist of the following. Figure S1: photos of Atacama Desert *Equisetum xylochaetum*. Figure S2: SILVAngs Classification Project information. Figure S3: bacterial genera detected in contigs containing 16S or 23S rDNA sequences at a mean sequencing depth >10× from *Equisetum xylochaetum* sampled at the less disturbed HUA site and/or more disturbed CHI site. Figure S4: bacterial genera detected from unassembled reads classifying as 16S or 23S rDNA sequences of at least ten reads from *Equisetum xylochaetum* sampled at the less disturbed HUA site and/or more disturbed CHI site. Figure S5: graphical representations of relative abundances of bacterial genera and phyla detected by at least ten reads from unassembled sequences classifying as 16S or 23S from *Equisetum xylochaetum* metagenomic sequence obtained for the less disturbed HUA site and more disturbed CHI site. Figure S6: bacterial genera detected by at least 50 reads classifying as 16S or 23S from *Equisetum xylochaetum* metagenomic sequence obtained for the less disturbed HUA site and more disturbed CHI site. Figure S7: contigs containing full-length genes associated with sulfur cycling and denitrification in *Equisetum xylochaetum* microbiomes at two sites differing in accessibility. Figure S8: bacterial taxa classified from contig data that were not classifiable to genus based on the SILVAngs database accessed in 2019, but detected by 16S or 23S rRNA genes at a mean sequencing depth >10× from *Equisetum xylochaetum* sampled at the accessible CHI and remote HUA sites. Figure S9: krona displays showing SILVAngs classifications based on ribosomal markers SSU and LSU. Figure S10: KEGG analyses of nitrogen metabolism genes for the two study sites. Figure S11: KEGG analyses of sulfur metabolism genes for the two study sites. Figure S12: genes and relative abundances associated with nitrogen fixation inferred from contigs assembled from metagenomic data, most present on a single large contig of gammaproteobacterial origin.

mcac060_suppl_Supplementary_Figure_S1

mcac060_suppl_Supplementary_Figure_S2

mcac060_suppl_Supplementary_Figure_S3

mcac060_suppl_Supplementary_Figure_S4

mcac060_suppl_Supplementary_Figure_S5

mcac060_suppl_Supplementary_Figure_S6

mcac060_suppl_Supplementary_Figure_S7

mcac060_suppl_Supplementary_Figure_S8

mcac060_suppl_Supplementary_Figure_S9

mcac060_suppl_Supplementary_Figure_S10

mcac060_suppl_Supplementary_Figure_S11

mcac060_suppl_Supplementary_Figure_S12
