# Supplementary figures and images for: Nitrogen fixation and other biogeochemically important features of Atacama Desert giant horsetail plant microbiomes inferred from metagenomic contig analysis
Source: Ann Bot. 2022 May 9;130(1):65–75. doi: 10.1093/aob/mcac060 (PMC9295926; doi:10.1093/aob/mcac060)

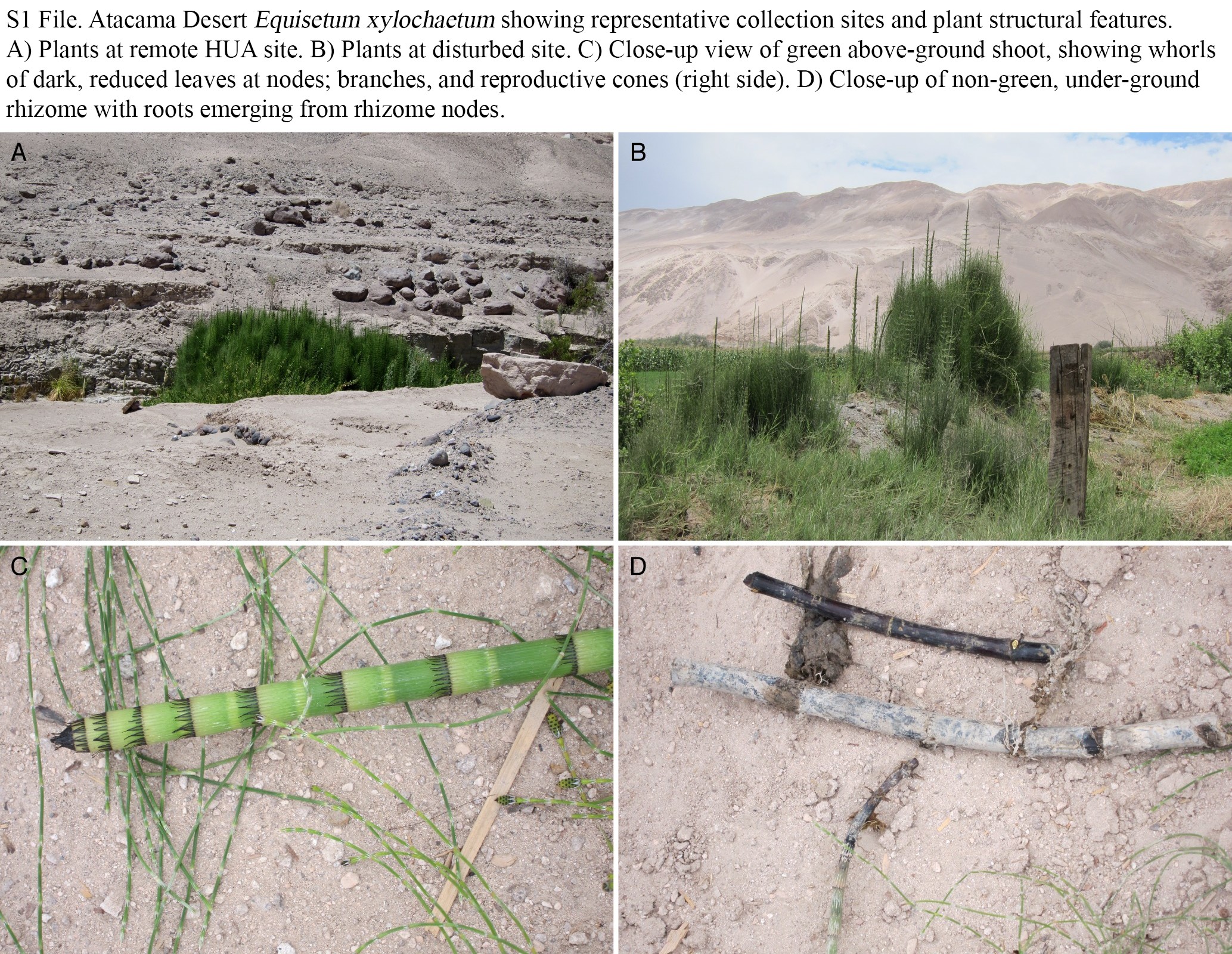

Supplement: mcac060_suppl_Supplementary_Figure_S1 [file mcac060_suppl_supplementary_figure_s1.jpeg]

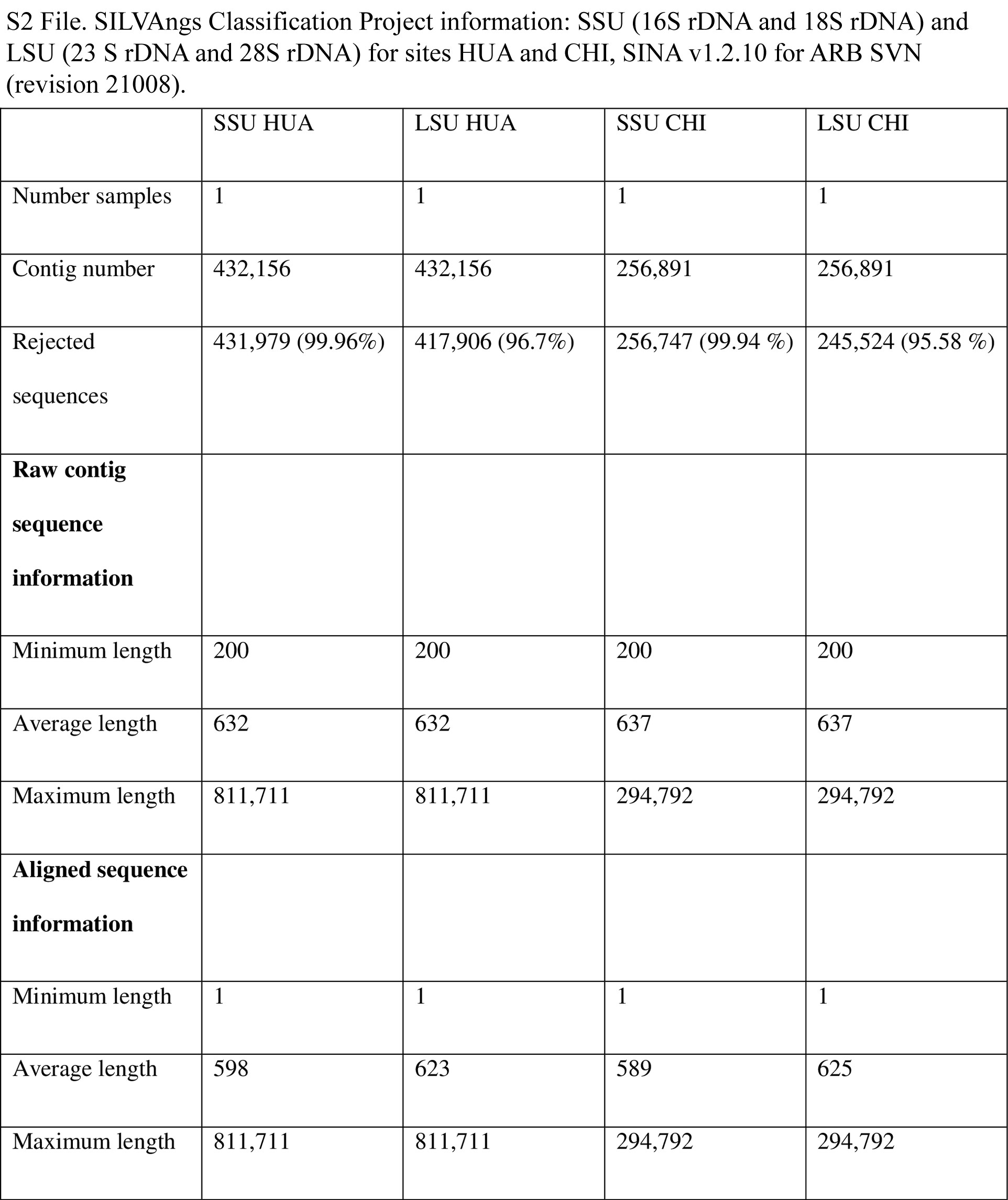

Supplement: mcac060_suppl_Supplementary_Figure_S2 [file mcac060_suppl_supplementary_figure_s2.jpeg]

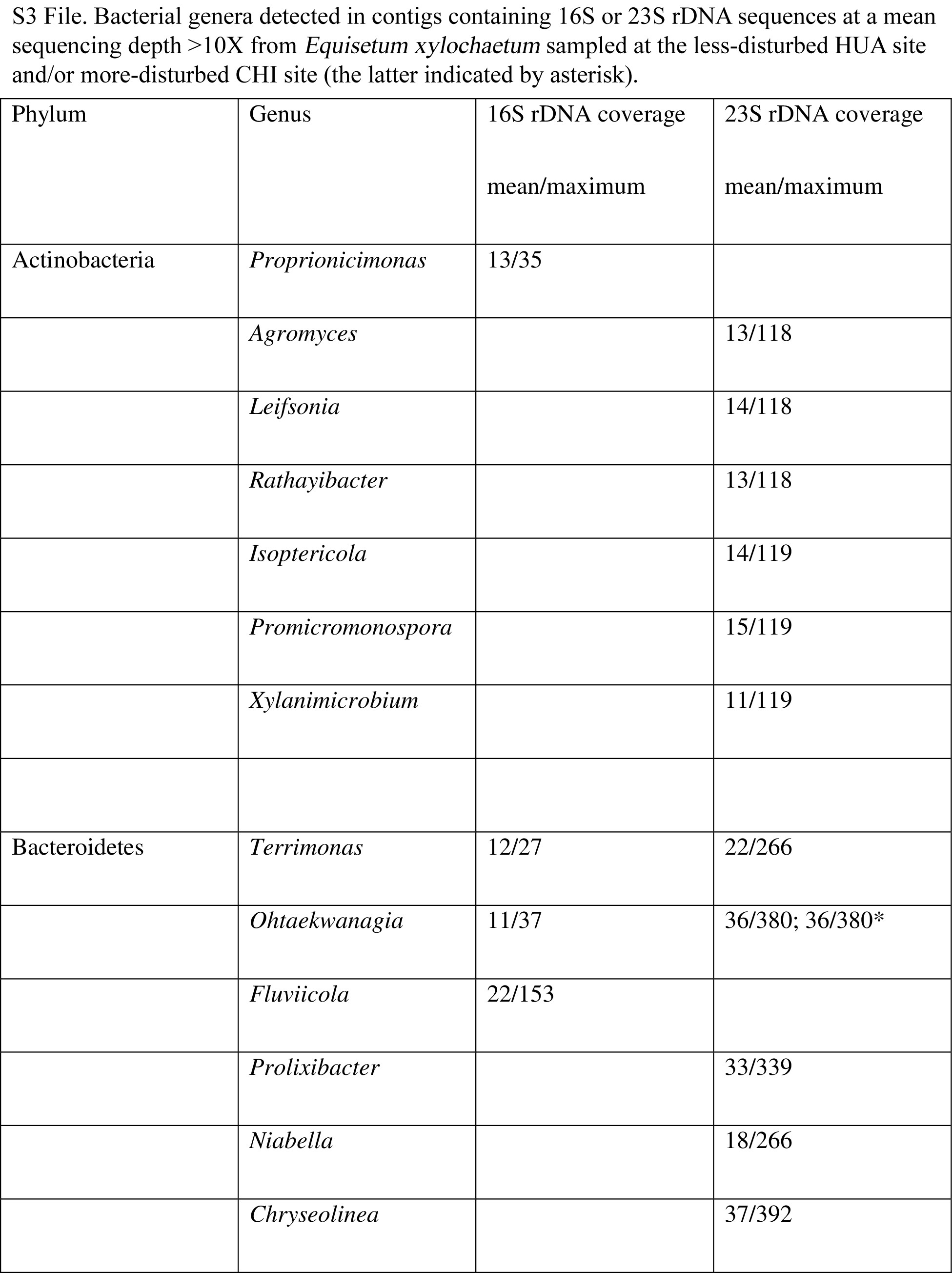

Supplement: mcac060_suppl_Supplementary_Figure_S3 [file mcac060_suppl_supplementary_figure_s3.jpeg]

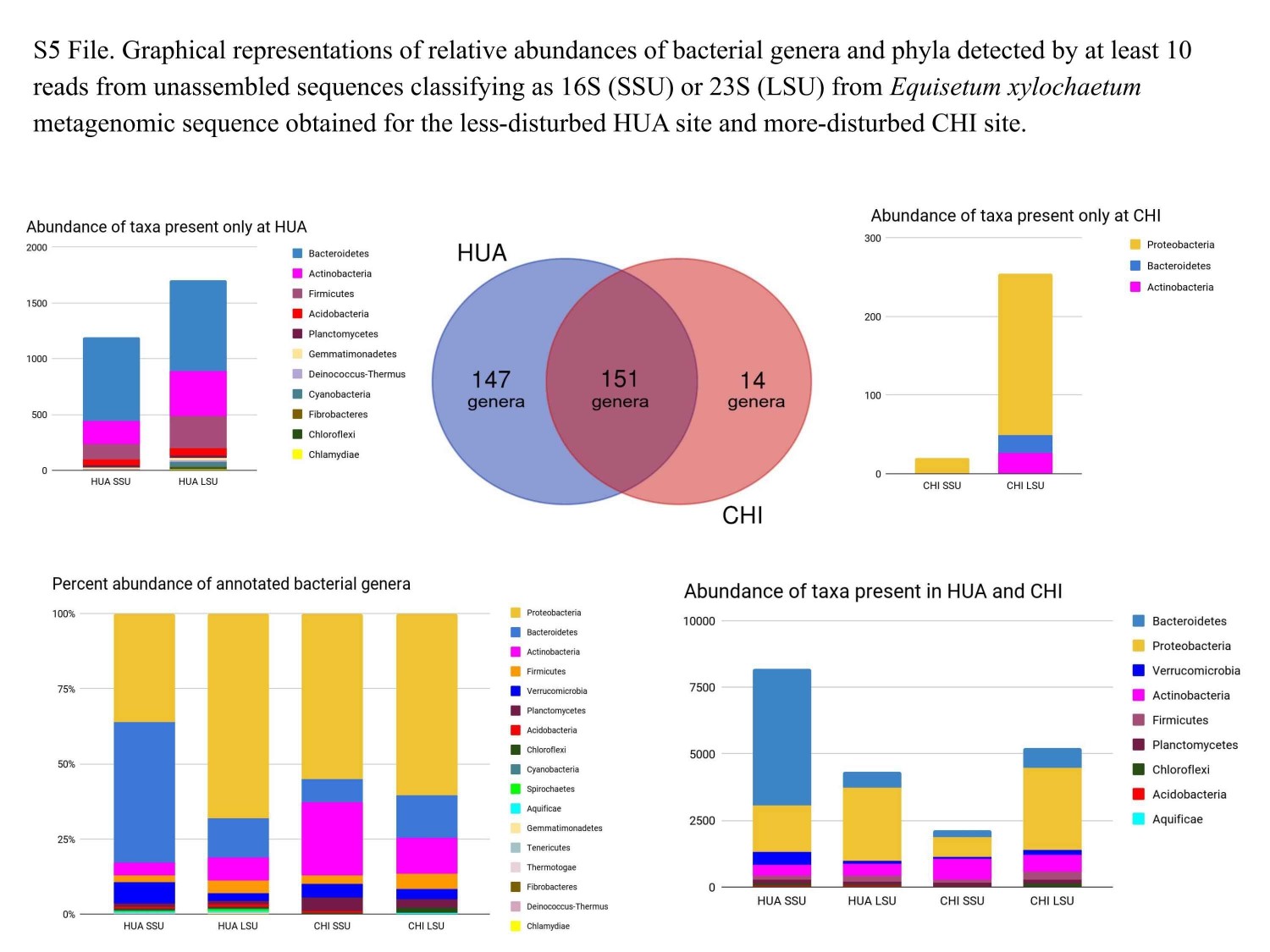

Supplement: mcac060_suppl_Supplementary_Figure_S5 [file mcac060_suppl_supplementary_figure_s5.jpeg]

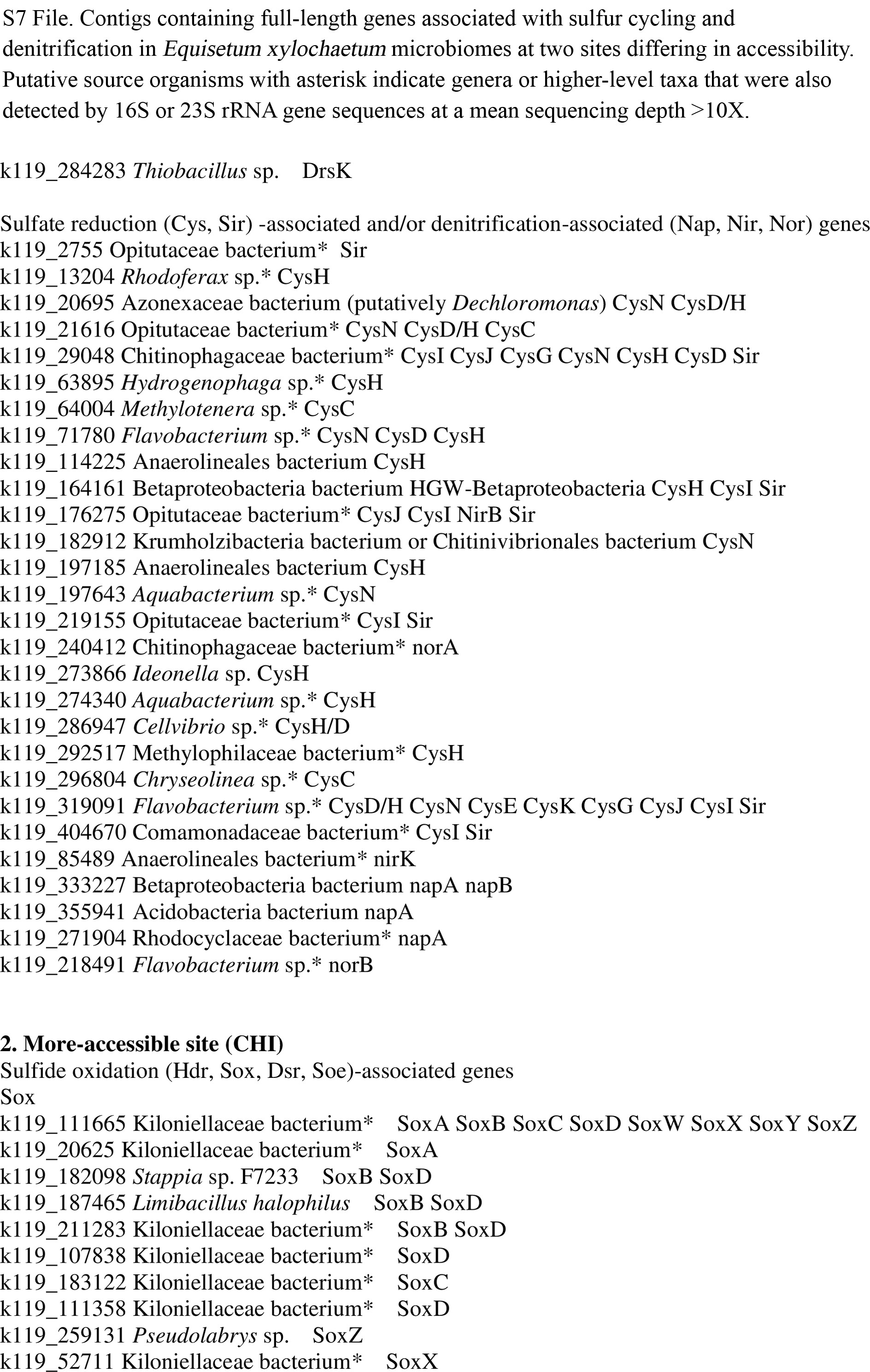

Supplement: mcac060_suppl_Supplementary_Figure_S7 [file mcac060_suppl_supplementary_figure_s7.jpeg]

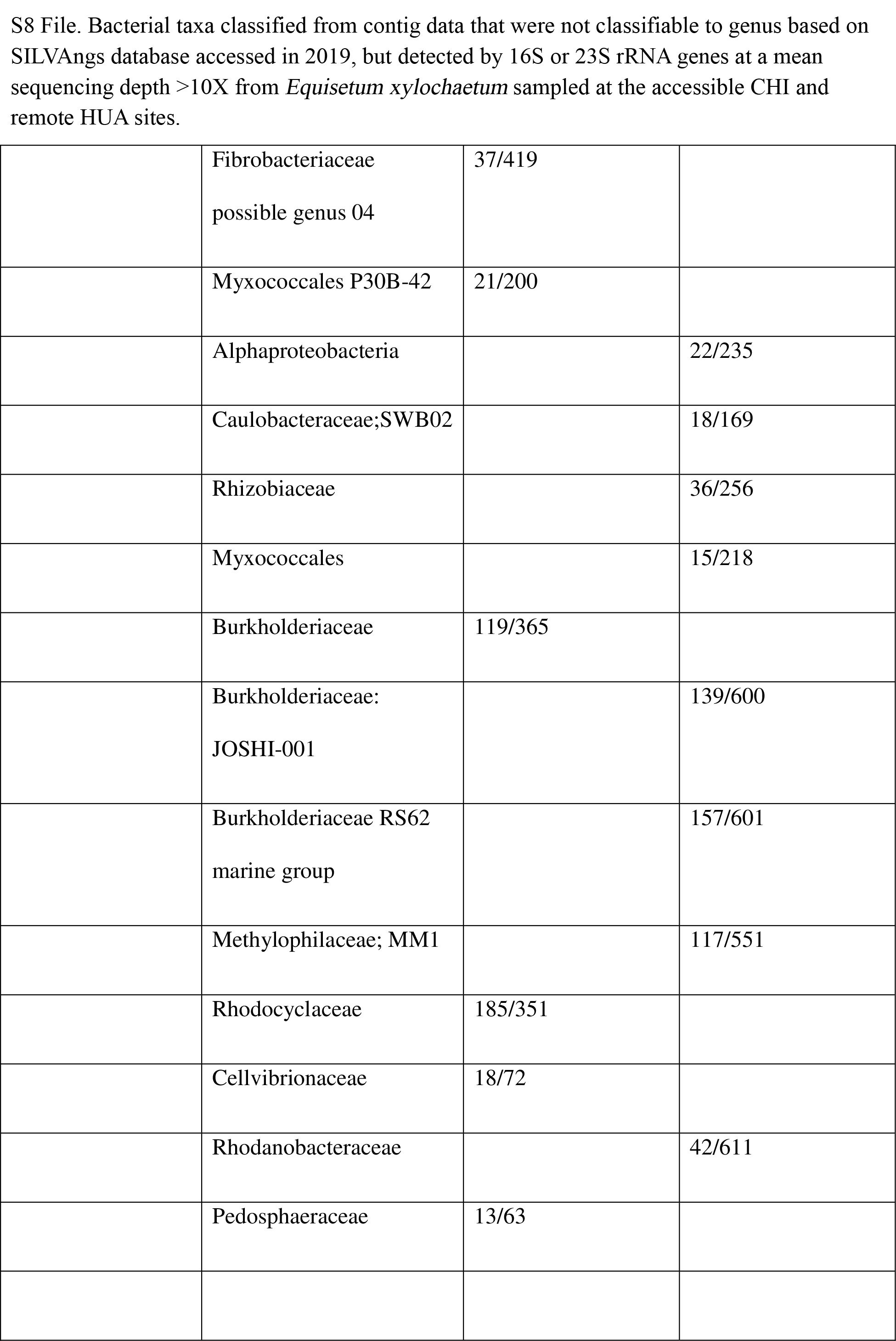

Supplement: mcac060_suppl_Supplementary_Figure_S8 [file mcac060_suppl_supplementary_figure_s8.jpeg]

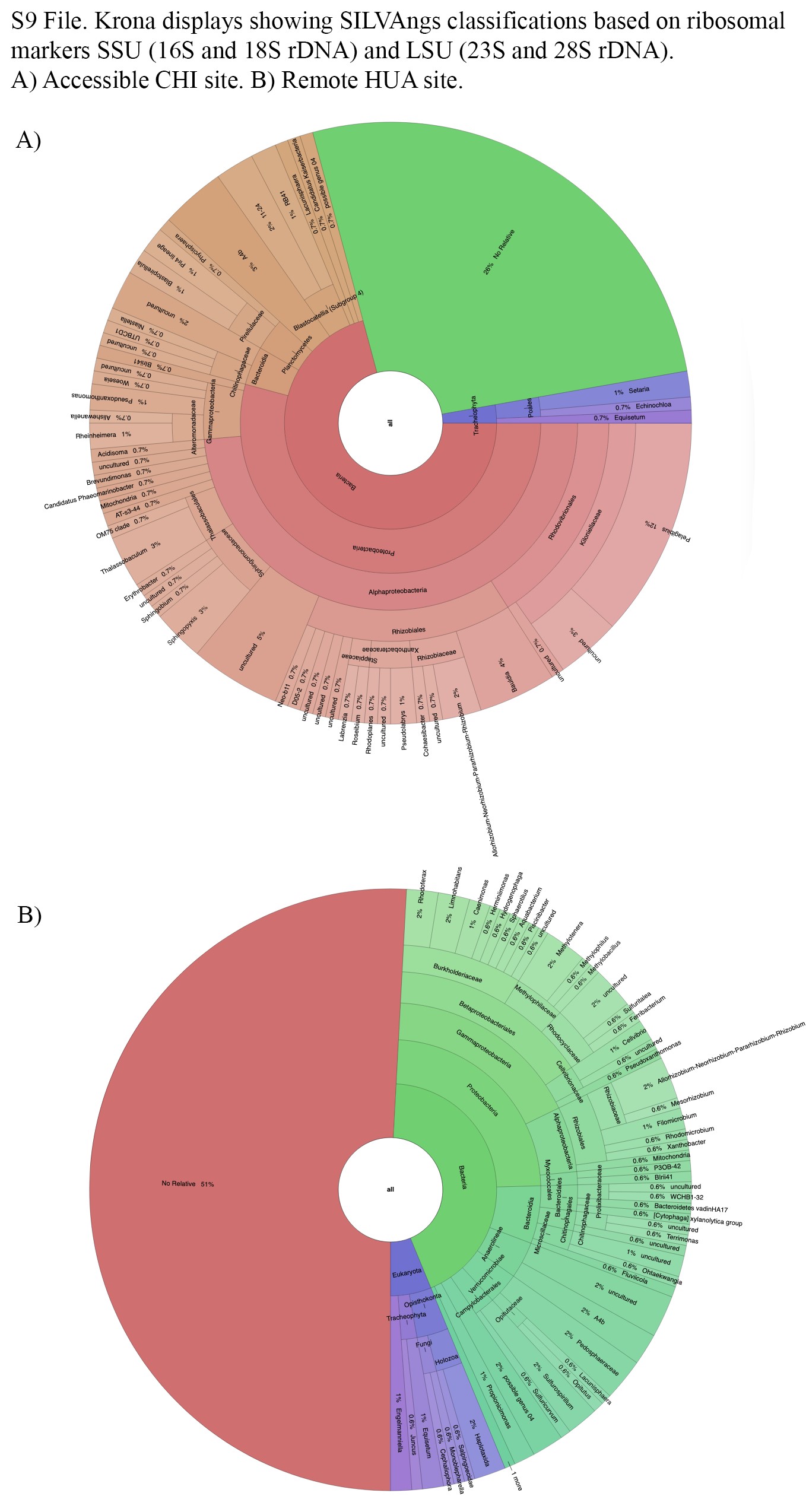

Supplement: mcac060_suppl_Supplementary_Figure_S9 [file mcac060_suppl_supplementary_figure_s9.jpeg]

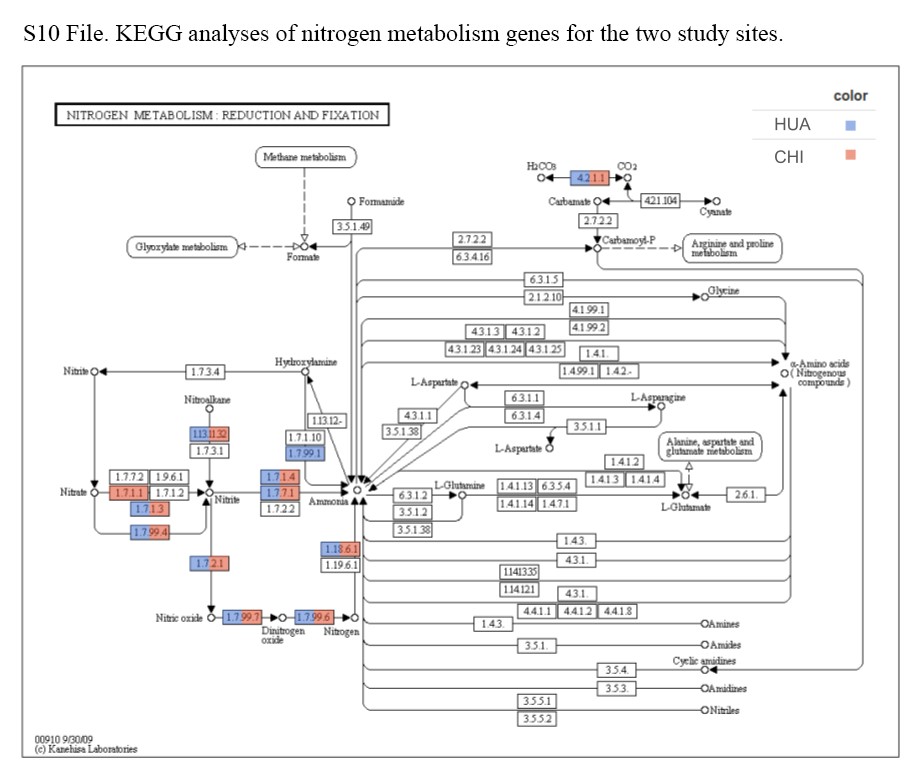

Supplement: mcac060_suppl_Supplementary_Figure_S10 [file mcac060_suppl_supplementary_figure_s10.jpeg]

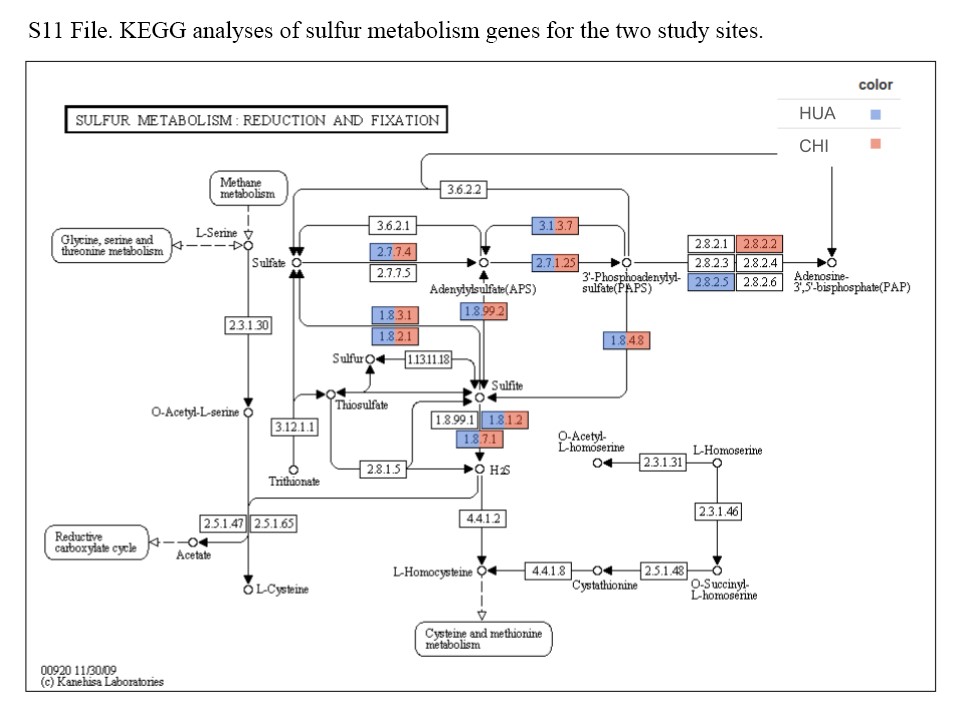

Supplement: mcac060_suppl_Supplementary_Figure_S11 [file mcac060_suppl_supplementary_figure_s11.jpeg]

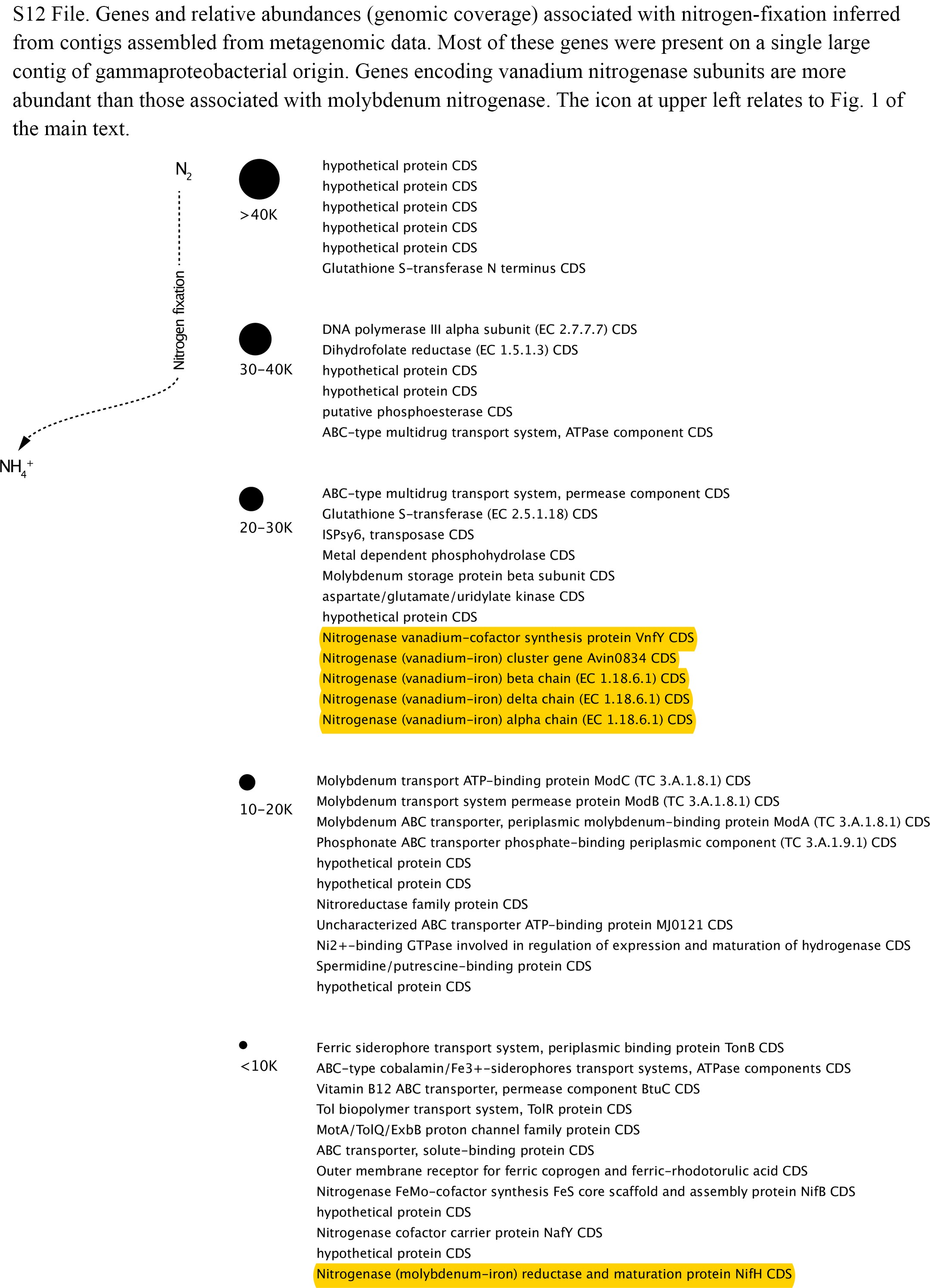

Supplement: mcac060_suppl_Supplementary_Figure_S12 [file mcac060_suppl_supplementary_figure_s12.jpeg]
