# Supplementary material for: Nitrogen fixation and other biogeochemically important features of Atacama Desert giant horsetail plant microbiomes inferred from metagenomic contig analysis
Source: Ann Bot. 2022 May 9;130(1):65–75. doi: 10.1093/aob/mcac060 (PMC9295926; doi:10.1093/aob/mcac060)
Supplement: mcac060_suppl_Supplementary_Figure_S4 [file mcac060_suppl_supplementary_figure_s4.pdf]

S4 File. Bacterial genera detected from unassembled reads classifying as 16S or 23S rDNA sequences of at least 10 reads from *Equisetum xylochaetum* sampled at the less-disturbed HUA site and/or more-distrubed CHI site.

		HUA (Abundance)		CHI (Abundance)	
Phylum	Genus	16S	23S	16S	23S
Acidobacteria	*Acidobacterium*	NA	25	NA	0
Acidobacteria	*Candidatus Solibacter*	42	22	0	0
Acidobacteria	*Candidatus Chloracidobacterium*	33	22	22	0
Acidobacteria	*Candidatus Koribacter*	12	22	0	0
Actinobacteria	*Acidimicrobium*	13	NA	0	NA
Actinobacteria	*Acidithiomicrobium*	NA	11	NA	0
Actinobacteria	*Actinomyces*	NA	19	NA	0
Actinobacteria	*Actinosynnema*	NA	32	NA	102
Actinobacteria	*Lentzea*	18	21	0	74
Actinobacteria	*Beutenbergia*	NA	22	NA	0
Actinobacteria	*Catenulispora*	NA	12	NA	0
Actinobacteria	*Cellulomonas*	NA	26	NA	0
Actinobacteria	*Corynebacterium*	24	19	0	0
Actinobacteria	*Frankia*	12	22	0	0
Actinobacteria	*Geodermatophilus*	18	28	0	0
Actinobacteria	*Gordonia*	NA	11	NA	0
Actinobacteria	*Janibacter*	NA	12	NA	0
Actinobacteria	*Tetrasphaera*	13	10	0	0
Actinobacteria	*Clavibacter*	NA	48	NA	0
Actinobacteria	*Cryobacterium*	25	19	0	0
Actinobacteria	*Leifsonia*	39	21	0	74
Actinobacteria	*Microbacterium*	65	16	0	0
Actinobacteria	*Okibacterium*	25	24	0	28
Actinobacteria	*Salinibacterium*	17	24	0	28
Actinobacteria	*Arthrobacter*	25	32	0	25
Actinobacteria	*Kocuria*	NA	0	NA	15
Actinobacteria	*Actinoplanes*	10	19	46	0
Actinobacteria	*Catenuloplanes*	0	12	103	0
Actinobacteria	*Dactylosporangium*	0	19	49	15
Actinobacteria	*Micromonospora*	12	16	438	0
Actinobacteria	*Salinispora*	15	24	72	28
Actinobacteria	*Mycobacterium*	32	16	16	0

		HUA (Abundance)		CHI (Abundance)	
Phylum	Genus	16S	23S	16S	23S
Actinobacteria	*Rhodococcus*	21	24	13	28
Actinobacteria	*Kribbella*	NA	10	NA	0
Actinobacteria	*Nocardioides*	20	24	0	28
Actinobacteria	*Promicromonospora*	44	24	0	28
Actinobacteria	*Xylanimonas*	NA	22	NA	0
Actinobacteria	*Microlunatus*	12	16	0	0
Actinobacteria	*Propionibacterium*	32	24	0	28
Actinobacteria	*Amycolatopsis*	0	32	33	102
Actinobacteria	*Sanguibacter*	NA	41	NA	0
Actinobacteria	*Streptomyces*	62	10	35	0
Actinobacteria	*Actinomadura*	NA	0	NA	11
Actinobacteria	*Atopobium*	14	32	0	25
Actinobacteria	*Eggerthella*	NA	13	NA	0
Actinobacteria	*Slackia*	16	24	0	28
Actinobacteria	*Conexibacter*	22	26	0	0
Aquificae	*Persephonella*	24	24	0	28
Bacteroidetes	*Bacteroides*	146	32	0	0
Bacteroidetes	*Butyricimonas*	10	22	0	0
Bacteroidetes	*Odoribacter*	10	24	0	28
Bacteroidetes	*Parabacteroides*	17	24	0	28
Bacteroidetes	*Porphyromonas*	35	24	0	28
Bacteroidetes	*Prevotella*	17	24	0	28
Bacteroidetes	*Alistipes*	25	32	0	102
Bacteroidetes	*Candidatus Azobacteroides*	35	22	0	0
Bacteroidetes	*Cyclobacterium*	10	19	0	0
Bacteroidetes	*Cytophaga*	223	19	43	15
Bacteroidetes	*Dyadobacter*	10	19	0	16
Bacteroidetes	*Flexibacter*	94	19	32	0
Bacteroidetes	*Hymenobacter*	28	21	19	74
Bacteroidetes	*Marinoscillum*	29	16	27	0
Bacteroidetes	*Microscilla*	61	16	19	0
Bacteroidetes	*Spirosoma*	NA	0	NA	23
Bacteroidetes	*Flexithrix*	49	19	32	0
Bacteroidetes	*Blattabacterium*	NA	15	NA	0
Bacteroidetes	*Arenibacter*	121	32	0	102

		HUA (Abundance)		CHI (Abundance)	
Phylum	Genus	16S	23S	16S	23S
Bacteroidetes	*Capnocytophaga*	1168	22	34	0
Bacteroidetes	*Cellulophaga*	70	26	0	0
Bacteroidetes	*Chryseobacterium*	NA	23	NA	11
Bacteroidetes	*Coenonia*	267	26	0	0
Bacteroidetes	*Elizabethkingia*	47	19	0	16
Bacteroidetes	*Flavobacterium*	1952	19	0	16
Bacteroidetes	*Gramella*	41	11	0	63
Bacteroidetes	*Kordia*	NA	12	NA	0
Bacteroidetes	*Leeuwenhoekiella*	365	21	0	74
Bacteroidetes	*Myroides*	135	16	0	0
Bacteroidetes	*Polaribacter*	NA	174	NA	0
Bacteroidetes	*Psychroflexus*	179	24	0	28
Bacteroidetes	*Riemerella*	193	24	0	28
Bacteroidetes	*Robiginitalea*	NA	247	NA	0
Bacteroidetes	*Tenacibaculum*	15	10	0	0
Bacteroidetes	*Zunongwangia*	NA	139	NA	0
Bacteroidetes	*Salinibacter*	14	24	0	28
Bacteroidetes	*Saprospira*	11	24	0	28
Bacteroidetes	*Pedobacter*	117	24	0	28
Bacteroidetes	*Sphingobacterium*	33	10	0	0
Bacteroidetes	*Chitinophaga*	38	26	11	0
Bacteroidetes	*Terrimonas*	169	10	34	0
Bacteroidetes	*Candidatus Amoebophilus*	13	22	0	0
Bacteroidetes	*Candidatus Cardinium*	15	22	0	0
Bacteroidetes	*Prolixibacter*	97	24	0	28
Chlamydiae	*Candidatus Fritschea*	NA	10	NA	0
Chloroflexi	*Roseiflexus*	NA	12	NA	0
Chloroflexi	*Herpetosiphon*	46	21	0	74
Chloroflexi	*Dehalococcoides*	30	19	12	15
Cyanobacteria	*Nodularia*	NA	10	NA	0
Cyanobacteria	*Leptolyngbya*	NA	20	NA	0
Cyanobacteria	*Prochlorococcus*	NA	12	NA	0
Deinococcus-Thermus	*Thermus*	14	10	0	0
Fibrobacteres	*Fibrobacter*	NA	13	NA	0

		HUA (Abundance)		CHI (Abundance)	
Phylum	Genus	16S	23S	16S	23S
Firmicutes	*Alicyclobacillus*	NA	11	NA	0
Firmicutes	*Bacillus*	34	32	14	25
Firmicutes	*Geobacillus*	10	22	0	0
Firmicutes	*Exiguobacterium*	0	19	19	16
Firmicutes	*Enterococcus*	NA	15	NA	0
Firmicutes	*Lactobacillus*	37	21	21	74
Firmicutes	*Fructobacillus*	NA	188	NA	0
Firmicutes	*Streptococcus*	NA	12	NA	0
Firmicutes	*Alkaliphilus*	13	32	0	102
Firmicutes	*Clostridium*	122	26	0	0
Firmicutes	*Oxobacter*	0	24	11	28
Firmicutes	*Desulfotomaculum*	50	19	14	15
Firmicutes	*Ruminococcus*	16	24	0	28
Firmicutes	*Halothermothrix*	NA	10	NA	0
Firmicutes	*Veillonella*	0	39	12	0
Gemmatimonadetes	*Gemmatimonas*	14	22	0	0
Planctomycetes	*Blastopirellula*	24	22	53	0
Planctomycetes	*Gemmata*	17	22	0	0
Planctomycetes	*Isosphaera*	17	21	0	74
Planctomycetes	*Pirellula*	30	24	33	28
Planctomycetes	*Planctomyces*	45	24	34	28
Planctomycetes	*Rhodopirellula*	23	24	22	28
Proteobacteria	*Brevundimonas*	NA	93	NA	12
Proteobacteria	*Caulobacter*	13	12	0	0
Proteobacteria	*Phenylobacterium*	NA	71	NA	48
Proteobacteria	*Fulvimarina*	NA	19	NA	23
Proteobacteria	*Bartonella*	16	32	37	0
Proteobacteria	*Beijerinckia*	17	32	12	0
Proteobacteria	*Bradyrhizobium*	17	22	0	0
Proteobacteria	*Nitrobacter*	NA	10	NA	0
Proteobacteria	*Oligotropha*	NA	19	NA	0
Proteobacteria	*Rhodopseudomonas*	25	24	0	28
Proteobacteria	*Brucella*	76	22	17	0
Proteobacteria	*Mycoplana*	NA	0	NA	17
Proteobacteria	*Ochrobactrum*	0	24	33	28

		HUA (Abundance)		CHI (Abundance)	
Phylum	Genus	16S	23S	16S	23S
Proteobacteria	*Hyphomicrobium*	12	21	0	74
Proteobacteria	*Methylobacterium*	NA	42	NA	56
Proteobacteria	*Methylocystis*	0	16	16	0
Proteobacteria	*Methylosinus*	NA	26	NA	19
Proteobacteria	*Chelativorans*	NA	100	NA	89
Proteobacteria	*Hoeflea*	NA	53	NA	18
Proteobacteria	*Mesorhizobium*	39	16	42	0
Proteobacteria	*Parvibaculum*	NA	40	NA	126
Proteobacteria	*Phyllobacterium*	16	24	72	28
Proteobacteria	*Agrobacterium*	45	32	17	102
Proteobacteria	*Candidatus Liberibacter*	0	22	12	0
Proteobacteria	*Ensifer*	NA	29	NA	18
Proteobacteria	*Rhizobium*	146	24	76	28
Proteobacteria	*Shinella*	NA	39	NA	20
Proteobacteria	*Sinorhizobium*	NA	96	NA	109
Proteobacteria	*Azorhizobium*	NA	0	NA	25
Proteobacteria	*Xanthobacter*	NA	117	NA	74
Proteobacteria	*Hirschia*	NA	18	NA	24
Proteobacteria	*Hyphomonas*	19	21	0	74
Proteobacteria	*Maricaulis*	NA	63	NA	60
Proteobacteria	*Jannaschia*	11	21	0	74
Proteobacteria	*Labrenzia*	0	21	11	74
Proteobacteria	*Oceanicola*	NA	34	NA	47
Proteobacteria	*Octadecabacter*	NA	0	NA	11
Proteobacteria	*Pannonibacter*	0	24	25	28
Proteobacteria	*Paracoccus*	10	24	55	28
Proteobacteria	*Pseudovibrio*	NA	19	NA	24
Proteobacteria	*Rhodobacter*	30	24	10	28
Proteobacteria	*Roseobacter*	0	24	55	28
Proteobacteria	*Stappia*	0	10	12	0
Proteobacteria	*Thalassobium*	NA	0	NA	10
Proteobacteria	*Acetobacter*	0	NA	20	NA
Proteobacteria	*Acidiphilium*	NA	0	NA	20
Proteobacteria	*Asaia*	NA	0	NA	14
Proteobacteria	*Gluconacetobacter*	0	28	16	0

		HUA (Abundance)		CHI (Abundance)	
Phylum	Genus	16S	23S	16S	23S
Proteobacteria	*Gluconobacter*	NA	11	NA	63
Proteobacteria	*Granulibacter*	NA	21	NA	74
Proteobacteria	*Roseomonas*	NA	24	NA	18
Proteobacteria	*Azospirillum*	45	32	21	25
Proteobacteria	*Magnetospirillum*	12	16	0	0
Proteobacteria	*Rhodospirillum*	NA	64	NA	192
Proteobacteria	*Ehrlichia*	NA	0	NA	10
Proteobacteria	*Wolbachia*	NA	0	NA	10
Proteobacteria	*Rickettsia*	NA	23	NA	11
Proteobacteria	*Caedibacter*	NA	19	NA	0
Proteobacteria	*Erythrobacter*	NA	22	NA	0
Proteobacteria	*Novosphingobium*	35	24	0	28
Proteobacteria	*Sphingobium*	21	10	10	0
Proteobacteria	*Sphingomonas*	30	10	0	0
Proteobacteria	*Sphingopyxis*	0	10	14	0
Proteobacteria	*Zymomonas*	NA	13	NA	0
Proteobacteria	*Bordetella*	NA	47	NA	0
Proteobacteria	*Derxia*	31	19	0	15
Proteobacteria	*Taylorella*	NA	18	NA	0
Proteobacteria	*Burkholderia*	204	22	0	0
Proteobacteria	*Cupriavidus*	NA	62	NA	0
Proteobacteria	*Limnobacter*	NA	38	NA	0
Proteobacteria	*Ralstonia*	NA	78	NA	0
Proteobacteria	*Acidovorax*	NA	743	NA	0
Proteobacteria	*Albidiferax*	52	32	0	102
Proteobacteria	*Comamonas*	NA	45	NA	0
Proteobacteria	*Curvibacter*	NA	430	NA	0
Proteobacteria	*Delftia*	NA	29	NA	0
Proteobacteria	*Polaromonas*	NA	183	NA	0
Proteobacteria	*Variovorax*	16	39	0	0
Proteobacteria	*Verminephrobacter*	NA	169	NA	0
Proteobacteria	*Xenophilus*	12	17	0	0
Proteobacteria	*Herminiimonas*	13	21	0	74
Proteobacteria	*Oxalobacter*	NA	17	NA	0
Proteobacteria	*Aquabacterium*	169	32	0	102

		HUA (Abundance)		CHI (Abundance)	
Phylum	Genus	16S	23S	16S	23S
Proteobacteria	*Leptothrix*	NA	406	NA	0
Proteobacteria	*Methylibium*	NA	475	NA	0
Proteobacteria	*Thiomonas*	NA	66	NA	0
Proteobacteria	*Sideroxydans*	NA	229	NA	0
Proteobacteria	*Thiobacillus*	15	10	0	0
Proteobacteria	*Methylobacillus*	11	16	0	0
Proteobacteria	*Methylotenera*	14	16	0	0
Proteobacteria	*Methylovorus*	NA	226	NA	0
Proteobacteria	*Eikenella*	14	19	0	16
Proteobacteria	*Laribacter*	NA	34	NA	0
Proteobacteria	*Lutiella*	NA	11	NA	0
Proteobacteria	*Neisseria*	254	24	0	28
Proteobacteria	*Nitrosomonas*	NA	58	NA	0
Proteobacteria	*Nitrosospira*	NA	147	NA	0
Proteobacteria	*Aromatoleum*	NA	41	NA	0
Proteobacteria	*Azoarcus*	20	32	0	25
Proteobacteria	*Azospira*	41	32	0	25
Proteobacteria	*Dechloromonas*	229	19	0	15
Proteobacteria	*Thauera*	155	10	0	0
Proteobacteria	*Candidatus Accumulibacter*	NA	118	NA	0
Proteobacteria	*Kinetoplastibacterium*	20	21	10	74
Proteobacteria	*Bdellovibrio*	NA	15	NA	0
Proteobacteria	*Desulfobacterium*	NA	26	NA	15
Proteobacteria	*Desulfococcus*	NA	13	NA	0
Proteobacteria	*Desulfosarcina*	59	19	0	15
Proteobacteria	*Desulfotalea*	NA	48	NA	0
Proteobacteria	*Desulfovibrio*	12	19	12	15
Proteobacteria	*Desulfurella*	26	19	0	15
Proteobacteria	*Geobacter*	NA	72	NA	38
Proteobacteria	*Pelobacter*	NA	104	NA	0
Proteobacteria	*Stigmatella*	78	10	0	0
Proteobacteria	*Haliangium*	NA	40	NA	10
Proteobacteria	*Anaeromyxobacter*	23	32	0	102
Proteobacteria	*Corallococcus*	12	26	0	0
Proteobacteria	*Myxococcus*	NA	26	NA	0

		HUA (Abundance)		CHI (Abundance)	
Phylum	Genus	16S	23S	16S	23S
Proteobacteria	*Nannocystis*	NA	23	NA	18
Proteobacteria	*Chondromyces*	0	26	15	0
Proteobacteria	*Sorangium*	20	10	35	0
Proteobacteria	*Syntrophus*	NA	34	NA	0
Proteobacteria	*Syntrophobacter*	NA	38	NA	0
Proteobacteria	*Arcobacter*	NA	23	NA	0
Proteobacteria	*Sulfurospirillum*	12	10	0	0
Proteobacteria	*Helicobacter*	NA	28	NA	10
Proteobacteria	*Sulfurimonas*	NA	0	NA	73
Proteobacteria	*Acidithiobacillus*	11	NA	0	NA
Proteobacteria	*Aeromonas*	NA	44	NA	50
Proteobacteria	*Tolumonas*	NA	13	NA	0
Proteobacteria	*Anaerobiospirillum*	NA	13	NA	0
Proteobacteria	*Alteromonas*	NA	0	NA	15
Proteobacteria	*Marinobacter*	NA	12	NA	0
Proteobacteria	*Saccharophagus*	NA	17	NA	29
Proteobacteria	*Moritella*	NA	10	NA	48
Proteobacteria	*Pseudoalteromonas*	NA	13	NA	17
Proteobacteria	*Shewanella*	22	24	0	28
Proteobacteria	*Teredinibacter*	11	10	0	0
Proteobacteria	*Allochromatium*	NA	15	NA	0
Proteobacteria	*Alkalilimnicola*	NA	22	NA	18
Proteobacteria	*Thioalkalivibrio*	23	10	0	0
Proteobacteria	*Buchnera*	NA	15	NA	0
Proteobacteria	*Dickeya*	0	19	12	15
Proteobacteria	*Erwinia*	NA	11	NA	0
Proteobacteria	*Escherichia*	NA	11	NA	0
Proteobacteria	*Coxiella*	11	19	0	0
Proteobacteria	*Legionella*	11	21	0	74
Proteobacteria	*Methylococcus*	NA	24	NA	0
Proteobacteria	*Methylomonas*	39	16	0	0
Proteobacteria	*Alcanivorax*	NA	37	NA	0
Proteobacteria	*Kangiella*	NA	10	NA	0
Proteobacteria	*Hahella*	13	21	0	74
Proteobacteria	*Halomonas*	NA	21	NA	0

		HUA (Abundance)		CHI (Abundance)	
Phylum	Genus	16S	23S	16S	23S
Proteobacteria	*Bermanella*	NA	10	NA	0
Proteobacteria	*Marinomonas*	NA	21	NA	0
Proteobacteria	*Neptuniibacter*	NA	39	NA	0
Proteobacteria	*Haemophilus*	NA	17	NA	0
Proteobacteria	*Acinetobacter*	NA	32	NA	19
Proteobacteria	*Azotobacter*	NA	16	NA	0
Proteobacteria	*Cellvibrio*	11	26	0	0
Proteobacteria	*Pseudomonas*	104	24	37	28
Proteobacteria	*Francisella*	15	19	0	0
Proteobacteria	*Methylophaga*	NA	14	NA	0
Proteobacteria	*Reinekea*	NA	15	NA	0
Proteobacteria	*Photobacterium*	NA	13	NA	17
Proteobacteria	*Vibrio*	NA	325	NA	36
Proteobacteria	*Aquimonas*	14	32	0	102
Proteobacteria	*Lysobacter*	50	16	0	0
Proteobacteria	*Stenotrophomonas*	NA	27	NA	0
Proteobacteria	*Xanthomonas*	41	17	49	0
Proteobacteria	*Xylella*	NA	22	NA	0
Proteobacteria	*Magnetococcus*	NA	26	NA	0
Proteobacteria	*Mariprofundus*	17	16	0	0
Spirochaetes	*Brachyspira*	NA	10	NA	0
Spirochaetes	*Leptospira*	NA	20	NA	0
Spirochaetes	*Spirochaeta*	36	10	0	0
Spirochaetes	*Treponema*	30	10	0	0
Tenericutes	*Candidatus Phytoplasma*	13	22	0	0
Tenericutes	*Mycoplasma*	NA	22	NA	0
Thermotogae	*Kosmotoga*	NA	11	NA	0
Thermotogae	*Thermosipho*	19	10	0	0
Verrucomicrobia	*Coraliomargarita*	11	26	0	0
Verrucomicrobia	*Opitutus*	336	24	35	28
Verrucomicrobia	*Chthoniobacter*	34	26	0	0
Verrucomicrobia	*Methylacidiphilum*	35	16	0	0
Verrucomicrobia	*Akkermansia*	50	32	10	102
Verrucomicrobia	*Prosthecobacter*	18	24	0	28
Verrucomicrobia	*Rubritalea*	30	24	0	28

		HUA (Abundance)		CHI (Abundance)	
Phylum	Genus	16S	23S	16S	23S
Verrucomicrobia	*Verrucomicrobium*	59	17	16	0
