# Supplementary material for: Nitrogen fixation and other biogeochemically important features of Atacama Desert giant horsetail plant microbiomes inferred from metagenomic contig analysis
Source: Ann Bot. 2022 May 9;130(1):65–75. doi: 10.1093/aob/mcac060 (PMC9295926; doi:10.1093/aob/mcac060)
Supplement: mcac060_suppl_Supplementary_Figure_S6 [file mcac060_suppl_supplementary_figure_s6.pdf]

S6 File. Bacterial genera detected by at least 50 reads classifying as 16S (SSU) or 23S (LSU) from *Equisetum xylochaetum* metagenomic sequence obtained for the less-disturbed HUA site and more-distrubed CHI site.

50 only HUA	50 HUA and CHI	50 only CHI
Actinobacteria	Proteobacteria	Actinobacteria
Microbacterium	*Xanthobacter*	*Actinosynnema*
Streptomyces	*Chelativorans*	*Lentzea*
		Leifsonia
Bacteroidetes	Proteobacteria	*Catenuloplanes*
Bacteroides	*Sinorhizobium*	*Micromonospora*
Cytophaga	*Rhodospirillum*	*Salinispora*
Flexibacter	*Maricaulis*	*Amycolatopsis*
Microscilla	*Leeuwenhoekiella*	
Capnocytophaga	*Aquabacterium*	Bacteroidetes
Cellulophaga	*Rhizobium*	Alistipes
Coenonia	*Arenibacter*	Hymenobacter
Flavobacterium	*Albidiferax*	Gramella
Myroides	*Akkermansia*	
Polaribacter		Chloroflexi
Psychroflexus		Herpetosiphon
Riemerella		
Robiginitalea		Firmicutes
Zunongwangia		*Alkaliphilus*
Pedobacter		*Lactobacillus*
Terrimonas		
Prolixibacter		Planctomycetes
		Blastopirellula
Firmicutes		*Isosphaera*
Fructobacillus		
Clostridium		
Desulfotomaculum		

50 only HUA	50 HUA and CHI	50 only CHI
<u>Proteobacteria</u>		<u>Proteobacteria</u>
Brevundimonas		*Hyphomicrobium*
Phenylobacterium		*Methylobacterium*
Brucella		*Parvibaculum*
Hoeflea		*Phyllobacterium*
Burkholderia		*Agrobacterium*
Cupriavidus		*Hyphomonas*
Ralstonia		*Jannaschia*
Acidovorax		*Labrenzia*
Curvibacter		*Paracoccus*
Polaromonas		*Roseobacter*
Verminephrobacter		*Gluconobacter*
Leptothrix		*Granulibacter*
Methylibium		*Herminiimonas*
Thiomonas		*Kinetoplastibacterium*
Sideroxydans		*Anaeromyxobacter*
Methylovorus		*Sulfurimonas*
Neisseria		*Aeromonas*
Nitrosomonas		*Legionella*
Nitrosospira		*Hahella*
Dechloromonas		*Aquimonas*
Thauera		
Candidatus Accumulibacter		
Desulfosarcina		
Geobacter		
Pelobacter		
Stigmatella		
Pseudomonas		
Vibrio		
Lysobacter		

50 only HUA	50 HUA and CHI	50 only CHI
Verrucomicrobia<br>*Opitutus*<br>*Verrucomicrobium*		
